# Epidemiology of Surgical Site Infections: Incidence and Risk Factors at Jimma University Specialized and Comprehensive Hospital, Ethiopia

**DOI:** 10.3390/antibiotics15020201

**Published:** 2026-02-12

**Authors:** Mulatu Gashaw, Bikila Alemu, Andreas Wieser, Rahel Tamrat, Assefa Legesse Sisay, Kira Elsbernd, Rebecca Kisch, Gemechu Abera, Gersam Abera, Demisew Amenu Sori, Esayas Kebede Gudina, Arne Kroidl

**Affiliations:** 1 School of Medical Laboratory Sciences, Jimma University, Jimma P.O. Box 378, Ethiopia; bikila.alemu@ju.edu.et (B.A.); rahel.tamrat@ju.edu.et (R.T.); 2 CIH^LMU^ Center for International Health, University Hospital, LMU Munich, Leopoldstrasse 5, 80802 Munich, Germany; akroidl@lrz.uni-muenchen.de; 3 Institute of Infectious Disease and Tropical Medicine, LMU University Hospital, 80802 Munich, Germany; wieser@mvp.lmu.de (A.W.); elsbernd.kira@med.uni-muenchen.de (K.E.); rebecca.kisch@med.uni-muenchen.de (R.K.); 4 Fraunhofer Institute for Translational Medicine and Pharmacology ITMP, Immunology, Infection and Pandemic Research IIP, Türkenstraße 87, 80799 Munich, Germany; 5 German Center for Infection Research (DZIF), 80802 Munich, Germany; 6 Max von Pettenkofer-Institute, Medical Faculty, Ludwig Maximilian University of Munich, Elisabeth-Winterhalter-Weg 6, 81377 Munich, Germany; 7 Department of Epidemiology, Jimma University, Jimma P.O. Box 378, Ethiopia; assefa.legesse@ju.edu.et; 8 Institute for Medical Information Processing, Biometry and Epidemiology (IBE), Faculty of Medicine, LMU Munich, 81377 Munich, Germany; 9 Department of Surgery, Jimma University, Jimma P.O. Box 378, Ethiopia; gemechu.negasa@edu.ju.et (G.A.); gersam.abera@ju.edu.et (G.A.); 10 Department of Gynecology and Obstetrics, Jimma University, Jimma P.O. Box 378, Ethiopia; demisew.amenu@ju.edu.et; 11 Department of Internal Medicine, Jimma University, Jimma P.O. Box 378, Ethiopia

**Keywords:** incidence, prevalence, surgical site infection, multi-drug resistance, extended spectrum beta-lactamase

## Abstract

**Background**: Surgical site infections (SSIs) are healthcare-associated infections that can occur following surgical procedures, either at admission or within 30 days post-discharge. This study aimed to assess the incidence and associated risk factors for superficial SSI at a Tertiary Hospital in Ethiopia. **Methods**: A longitudinal study was conducted among patients undergoing surgery at Jimma University Specialized and Comprehensive Hospital (JUSCH) from 1 June to 30 September 2022. Pus, wound swab, or abscess samples were inoculated on Blood and MacConkey Agar for culture. Bacterial isolates were identified using MALDI-TOF, antimicrobial susceptibility testing was performed using the Kirby–Bauer disc diffusion method, and the results were interpreted according to EUCAST 2022 breakpoints. Incidence rates, Kaplan–Meier analysis, extended Cox regression, and violin plots were utilized to analyze and present the findings. **Results**: Among 1205 participants, 629 (52.2%) were male, and the median age was 27 years (IQR: 16–40). The incidence of SSI was 9.2 per 1000 person-days. Most SSIs occurred during hospitalization (81.1%), and the remaining primarily developed within the first week post-discharge. The culture positivity rate was 72.7%, yielding 252 isolates comprising 36 bacterial species. The most frequently identified organisms were *E. coli* (22.2%), *Acinetobacter* (20.2%), and *Klebsiella* (14.7%). Over 67% of Gram-negative bacteria were ESBL producers. Age, gender, residence, hospital ward, surgery area, emergency surgery, longer hospitalization, and the number of staff attending the surgery were identified as important risk factors. **Conclusions**: This study revealed a high incidence of SSI during hospitalization, with significant proportion identified post-discharge. The high rates of multidrug-resistant Gram-negative pathogens underscore the urgent need for comprehensive infection prevention and control measures.

## 1. Introduction

Surgical site infection (SSI) is a type of healthcare-associated infection (HAI) that occurs at or near the surgical incision site and may disseminate to other organs following a surgical procedure, either during admission or within 30 days post-discharge [1]. SSIs are characterized by signs such as redness, swelling, warmth, pain, discharge, or abscess formation [2]. They can be categorized as superficial (involving the skin and subcutaneous tissue), deep (involving deeper tissues or organs), or organ/space (affecting any part of the body other than the incision site) [3].

SSIs are caused by various microorganisms, including bacteria, viruses, and fungi, which may originate from the patient’s flora or exogenous sources such as the surgical team, medical devices, or the hospital environment [2,4,5,6]. The most common bacterial etiologies include *S. aureus*, *E. coli*, *K. pneumoniae*, *P. aeruginosa*, *A. baumannii*, and *Enterococcus* species [7,8]. Inadequate infection prevention and control practices, limited access to effective antibiotics, and multidrug-resistant (MDR) strains are the major contributing factors to SSI and related complications [1,5,8]. Methicillin-resistant *S. aureus* and beta-lactam-resistant Gram-negative bacteria are significant causes of SSI and pose substantial challenges in infection management [8,9]. In low-income countries, SSI prevalence ranges from 12% to 31% [6,10,11]. In Ethiopia, reported SSI prevalence varies from 6.8% to 25%, with common isolates being *E. coli*, *S. aureus*, *P. aeruginosa*, and *Klebsiella* species. Over 80% of these isolates are MDR [8,12,13] and often fail to respond to commonly used antibiotic treatments.

High SSI rates are linked to numerous patient- and procedure-related risk factors [1,14]. Procedure-related factors include emergency surgery, delays in surgical intervention, anesthesia type, wound type, anatomical site of surgery, admission diagnosis, and admission ward [15,16]. Patient-related factors such as gender, compromised immunity, malnutrition, and lack of preoperative screening also increase susceptibility to SSIs [2,5,9]. Health system and environmental conditions such as limited access to clean water, inadequate sanitation, poor infection prevention and control practices, suboptimal sterilization of equipment, and lack of adherence to aseptic measures further contribute to SSI risk [13,17]. High rates of MDR pathogens, overcrowded healthcare facilities, and broader socioeconomic factors such as poverty and limited healthcare resources also increase vulnerability to SSI [2,5].

In resource-limited settings, inconsistent SSI reporting and characterization make it difficult to capture their true burden [2]. Inadequate healthcare infrastructure, limited surveillance systems, and poor data collection practices contribute to this issue [6,10]. Variations in diagnostic practices, inconsistent SSI definitions, and a shortage of trained personnel further compromise data quality and comparability across studies [6]. Together, these factors limit the understanding of SSI epidemiology and impede the development of evidence-based interventions [18]. Although several studies on SSIs have been conducted in Ethiopia, the overall burden is not fully elucidated due to methodological limitations. Most of the studies included only certain procedures (e.g., cesarean section) or certain age groups (e.g., adult patients) and did not include post-discharge follow-up [8,19]. This study aimed to assess the incidence, bacterial profile, and associated risk factors of SSI up to 28-days post-discharge at JUCSH, a tertiary hospital in Ethiopia.

## 2. Results

### 2.1. Socio-Demographic and Clinical Patient Characteristics

A total of 1205 participants, 52% (629) of whom were male, underwent surgical procedures at JMC and were recruited for the study. The median age was 27 years (IQR: 16–40), with a range of 20 days to 90 years. Over two-thirds of the patients, 68% (n = 822), were rural residents. Approximately 41% of the surgical procedures were emergency operations. Of all the procedures, 51% (n = 615) were performed on or near the abdominal area. Ceftriaxone was provided as prophylaxis for almost all patients, (98.5%) based on the national guideline and its affordability, with most doses given before the surgical procedure (Table 1). The median length of hospital stay post-procedure varied across wards, ranging from 1 to 86 days. The longest stays were observed in the pediatric ward, with a median of 11 days (IQR: 8–15), and in the gynecology and obstetrics ward, also with a median of 11 days (IQR: 5–20). In contrast, the shortest stays were recorded in the maternity ward, with a median of 4 days (IQR: 3–5) (Supplementary Figure S1).

### 2.2. Follow-Up Information

**Figure 1:**
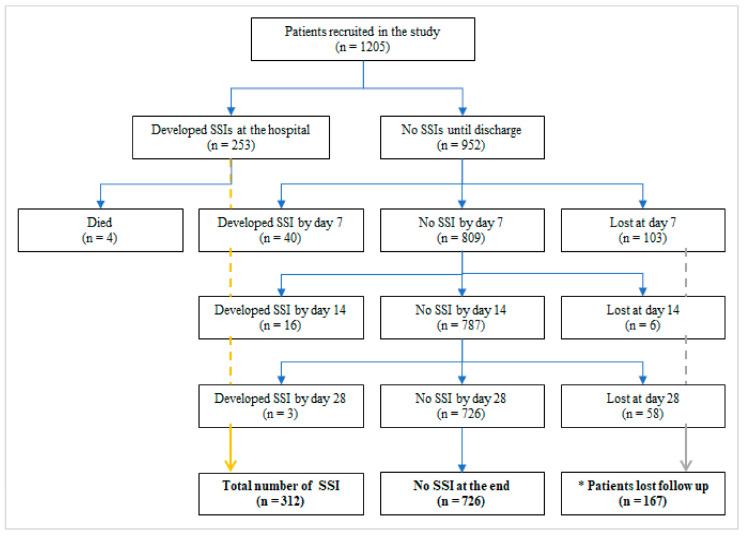
Study flow diagram of study participants. **Key**: * A total of 167 participants were lost to follow-up for various reasons. Specifically, 44 participants lacked contact phone numbers, 59 could not be reached by the research team, and 64 were not reachable during all scheduled calls for the oral clinical assessment. Consequently, total events were 312 and the total number of censored cases 167 (167 lost to follow-up and 726 who did not experience an event by the end of the follow-up period).

Complete follow-up information, including during the hospital stay and post-discharge, was available for 86.1% (n = 1038) of participants. Post-discharge follow-up was incomplete for the remaining 13.9% (n = 167) of participants. Reasons for loss to follow-up included insufficient or incorrect contact information (n = 44), repeatedly unsuccessful tracing attempts (n = 59), or successful tracing but non-attendance of scheduled follow-up appointments (n = 64) for clinical assessments. The study flow of participants is shown in Figure 1. By the end of the follow-up period, 312 patients were identified as having developed SSI, resulting in a cumulative prevalence of 25.9% (95% CI: 23.5–28.4). Of these, 81.1% (n = 253) developed SSI during hospitalization, while the remaining 18.9% (n = 59) developed the infection post-discharge. Most post-discharge SSIs appeared within the first seven days, with additional cases identified up to day 14, and only a few cases up to day 28 (Figure 1).

### 2.3. Incidence of Surgical Site Infection

The study participants were followed for a total of 33,866 person-days. The overall incidence rate of SSI was 9.2 per 1000 person-days (95% CI: 8.3–10.3). Incidence rates varied across inpatient units. The orthopedics ward exhibited the highest incidence at 20.8 per 1000 person-days (95% CI: 17.6–24.5), while the oral and maxillofacial surgery (OMFS) ward had the lowest incidence at 1.5 per 1000 person-days (95% CI: 0.4–6.2). The overall incidence of SSI post-discharge was 2.7 per 1000 person-days. Notably, most of these infections occurred during the first week following discharge, with a particularly high post-discharge incidence observed among maternity and pediatric participants. Additionally, the incidence rate was higher among male patients at 13.4 per 1000 person-days (95% CI: 11.8–15.3), as well as in patients who underwent surgical procedures near the head or neck at 16.9 per 1000 person-days (95% CI: 13.6–20.9), and in those who had surgeries on the abdomen at 11.8 per 1000 person-days (95% CI: 10.3–13.6) (Table 2).

### 2.4. Survival Estimate Using Kaplan–Meier Curve

**Figure 2:**
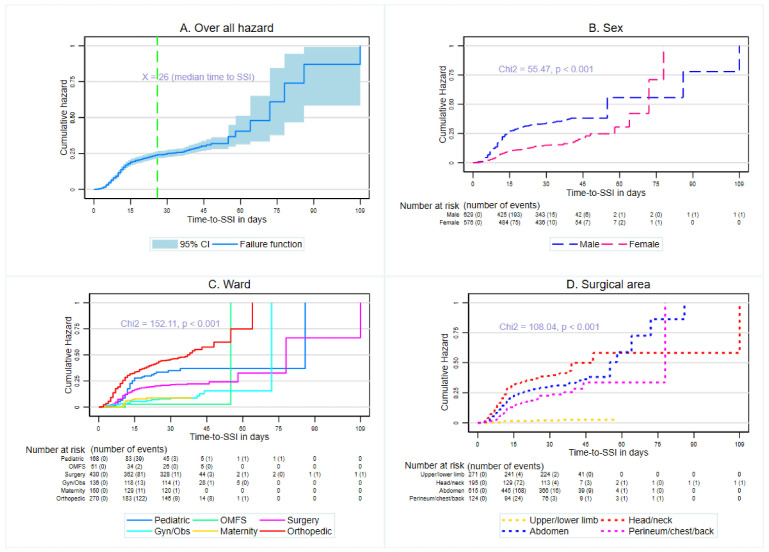
Kaplan–Meier analysis of time to SSI in study participants undergoing surgery at JMC.

The median time to develop SSI was 26 days (IQR: 12–38) (Figure 2A). Notably, significantly higher infection rates were found among patients admitted to orthopedic wards (Figure 2C), those undergoing head and neck surgeries (Figure 2D), and male patients (Figure 2B) (*p* < 0.001). This underscores the impact of both the clinical environment and surgical characteristics on the risk of infection.

### 2.5. Factors Associated with the Development of SSIs

Based on the extended Cox regression analysis, several risk factors were identified as significantly associated with the development of SSI. Patients aged 25–50 years had nearly twice the risk of developing SSI compared to those aged ≤14 years (aHR = 1.8; 95% CI: 1.21–2.71; *p* = 0.004), while patients aged ≥65 years had more than double the risk (aHR = 2.3; 95% CI: 1.21–4.24; *p* = 0.010). Furthermore, males were found to have 1.7 times greater risk of developing SSI compared to females (aHR = 1.7; 95% CI: 1.25–2.18; *p* < 0.001). Surgical patients who came from rural residences were 1.4 times more likely to develop SSI than those from urban residences (aHR = 1.4; 95% CI: 1.00–1.89; *p* = 0.049).

Hospital ward of admission and anatomical region of the procedure were also associated with increased risk of SSI. Patients admitted to Orthopedic wards had a 5.9 times higher risk of SSI compared to those in the Maternity ward (aHR = 5.9; 95% CI: 2.35–14.55; *p* < 0.001). Similarly, patients admitted to Surgery wards had 7.3 (aHR = 7.3; 95% CI: 1.82–29.05; *p* = 0.005) times higher risk of SSI compared to patients admitted to the Maternity ward. Surgery involving the head and neck, as well as abdominal procedures, was associated with significantly higher risks of SSI compared to procedures involving the limbs, with aHRs of 23.4 (95% CI: 10.10–54.34; *p* < 0.001) and 16.2 (95% CI: 7.10–37.08; *p* < 0.001), respectively. Procedures involving the perineum, chest, or back also carried a notably higher risk compared to surgeries involving the limbs (aHR = 11.0; 95% CI: 4.52–26.79; *p* < 0.001).

Patients who underwent emergency surgery had nearly twice the risk of SSI compared to those who had elective surgery (aHR = 1.9; 95% CI: 1.43–2.71; *p* < 0.001). Furthermore, the survival estimate revealed that each additional surgical staff member involved in the procedure was associated with a 2.2-fold increase in the hazard of developing SSI (aHR = 2.2; 95% CI: 1.29–3.65, *p* = 0.003). For every one day stay in the hospital, the hazard of SSI increased by 1% (aHR = 1.01; 95% CI: 1.00–1.02; *p* < 0.001) (Table 3).

### 2.6. Bacterial Profile of Surgical Site Infections

**Figure 3:**
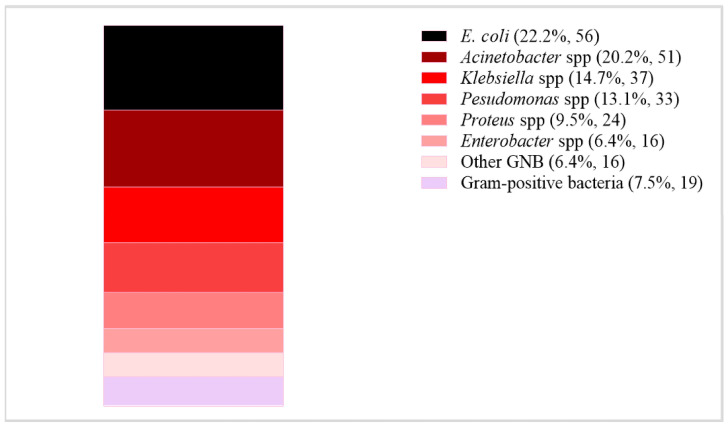
The frequency of bacteria isolated from samples collected from patients with suspected SSI. **Key**: ***Acinetobacter* spp.**: *A. baumannii*, *A. haemolyticus*, *A. pittii*, and *A. ursingii*. ***Klebsiella* spp.**: *K. oxytoca*, *K. pneumoniae*, and *K. variicola*. ***Pseudomonas* spp.**: *P. aeruginosa*, *P. mendocina*, *P. putida*, and other *Pseudomonas* species. ***Proteus* spp.**: *P. hauseri*, *P. mirabilis*, and *P. vulgaris*. ***Enterobacter* spp.**: *E. asburiae*, *E. cloacae*, and Other *Enterobacter* species. **Other GNB**: *C. freundii* (5), *P. rettgeri* (3), *L. adecarboxylata* (2), *M. morganii* (2), *Citrobacter* species (1), *S. marcescens* (1), *A. hydrophila* (1), and *S. maltophilia* (1). **Gram-positive bacteria**: *S. aureus* (7), *Corynebacterium striatum* (3), *S. cohnii* (2), *S. epidermidis* (2), *S. haemolyticus* (2), (1), *S. hominis* (1), *S. pasteuri* (1), and *S. warneri*.

Among the 312 patients who developed SSI, clinical samples such as pus, abscess, or wound swabs were obtained from 260 patients. Of these, 248 samples were collected during the primary hospitalization, while the remaining 12 were obtained during readmissions post-initial discharge. Of all clinical samples, 72.7% (189/260) were culture positive, with 4.2% (8/189) obtained from readmitted patients. In total, 252 isolates of 36 different bacteria species were identified from the culture-positive samples. Polymicrobial growth was observed in 31.2% (59/189) of the samples. The most frequently isolated bacteria were *E. coli* (56; 22.2%), followed by *Acinetobacter* (51; 20.2%) and *Klebsiella* species (37; 14.7%) (Figure 3).

### 2.7. Phenotypic AST Analysis

The analysis of antibiotic resistance patterns among Gram-negative bacterial isolates revealed alarming resistance trends for commonly used antibiotics. For instance, resistance to third-generation cephalosporins like cefotaxime and ceftazidime was observed in 86.7% and 68.7% of the Gram-negative isolates, respectively. Similarly, the resistance rates of fluoroquinolones among Gram-negative isolates ranged from 44% to 77%, which limits the effectiveness of these broad-spectrum antibiotics. Even carbapenems, which are considered last-resort antibiotics, showed resistance rates ranging from 9% in *E. coli* to 51% in *Acinetobacter* species. Further analysis indicated that more than 67% of all Gram-negative bacterial isolates were identified as ESBL producers. Furthermore, 78.5% were classified as multidrug-resistant according to the criteria defined by Magiorakos et al. in 2012 (Table 4) [20].

## 3. Discussion

The findings of this study underscore the substantial burden of SSI, with an overall prevalence of 25.9% and a cumulative incidence rate of 9.2 cases per 1000 person-days. Notably, we also observed high levels of antibiotic resistance among the bacterial pathogens isolated from these infections. Key risk factors identified included older patient age, male gender, rural residency, emergency procedures, surgery and orthopedic wards, longer hospitalization duration and specific surgical sites such as head, neck, abdomen, and pelvic area. These findings emphasize the need for targeted interventions to address the high burden of SSI and related poor outcomes. Moreover, the alarming prevalence of multidrug-resistant organisms, particularly among Gram-negative bacteria, compared with findings from studies in Western countries involving similar patient populations, underscores the urgent need for improved antibiotic stewardship and strengthened infection control practices to mitigate the growing threat of SSI in the study area and similar healthcare settings.

The incidence rate of SSI in the current study is comparable to previous studies conducted in Ethiopia which reported 11.7 cases per 1000 person-days, as well as a study in Kenya that reported 7.0 cases per 1000 person-days [12,21]. Moreover, the cumulative prevalence of SSI in this study is 25.9%. This is higher than previous studies from Ethiopia which reported 19.1% in Hawassa, 9.4% in Assella, and 6.8% in Lemlem Karl hospital [22,23,24]. Among all SSI cases, over 80% were observed during hospitalization. However, a substantial proportion of SSI cases occurred post-discharge, the majority of which manifested within the first one or two weeks. This highlights that focusing solely on SSI during the hospital stay provides an incomplete picture. Consequently, the findings underscore the critical importance of comprehensive surveillance and follow-up of patients beyond the initial hospital stay to optimize patient outcomes.

The overall culture positivity rate was 72.0% (189/260), which is consistent with previous studies conducted in Jimma (71.7%), Hawassa (71.1%), Mekelle (75%), and Gondar (69.7%) [25,26,27,28]. Unlike many previous studies in Ethiopia, the present study found that Gram-negative rods were the predominant pathogens responsible for SSI, accounting for 92.5% of all culture-confirmed cases. This difference may be attributed to the fact that most of the patients in this study underwent surgical procedures involving the abdominal area, increasing the likelihood of contamination from gastrointestinal contents, as demonstrated by a study from Tanzania [29]. Furthermore, the infections could have originated from the hospital environment, as the identified pathogens are commonly found in healthcare settings and are also frequently found on inanimate objects [30,31,32]. These findings highlight the critical need for rigorous environmental cleaning, disinfection protocols, and proper hand hygiene to reduce the risk of SSI [33].

The resistance profiles of the bacterial pathogens responsible for SSIs in this study revealed the presence of MDR strains. Notably, MDR isolates of *A. baumannii*, *K. pneumoniae*, *Pseudomonas* species, *Enterobacter* species, and *E. coli* were identified. These findings underscore the growing threat of AMR, which poses a substantial challenge in managing SSIs [9,34]. The results emphasize the urgent need for antimicrobial stewardship programs, careful and judicious use of antibiotics, and robust surveillance to detect and curb the spread of resistant bacteria. This is particularly critical in low-income countries, where access to alternative antibiotics for treating SSIs caused by these resistant pathogens is often limited [1,15,34].

The Kaplan–Meier and Cox-regression analysis in this study identified several risk factors associated with the development of SSI. These include being older age (>65 years), residing in a rural area, undergoing surgical procedures involving the abdomen, neck, head, or pelvis, admission to orthopedic or surgery wards, undergoing emergency surgery, higher number of surgical staff involved in the procedure, and longer hospital stay. However, these risks may be confounded by several other unmeasured but important factors, such as the complexity of the procedure and the time of the surgery. Nonetheless, targeting these physiological, clinical, personal, and environmental risk factors through targeted interventions could play a crucial role in reducing the incidence of SSI [35,36].

These findings also provide valuable insights for stakeholders to develop and implement effective infection prevention and control measures aimed at mitigating the burden of SSI [3]. For example, strategies such as preoperative patient screening and optimization, tailored perioperative management, meticulous surgical technique, and the appropriate use of antimicrobial prophylaxis can significantly contribute to reducing SSI rates. Implementing these measures is essential, especially in high-risk scenarios, to control and prevent the spread of infection [1,35].

### Strength and Limitation of the Study

Conducting a longitudinal study enables us to understand the dynamic nature of SSI over time, including its temporal trends and patterns, which provide a deeper understanding of how various factors influence SSI development over time. This approach is particularly valuable in identifying modifiable risk factors that can be targeted for intervention. Additionally, the study offers valuable insights into the epidemiology of SSIs in low-income countries, which can inform strategies to reduce the incidence.

However, several limitations should be considered. First, since this study was conducted at a single tertiary hospital, the findings may not be generalizable to other healthcare settings within the country or globally. Second, anaerobic bacteria were not investigated due to limited laboratory capability, leaving an important gap in understanding the contribution of these bacteria as etiologies of SSIs. Third, the proportion of post-discharge SSIs may not indicate the actual data, as patients with serious SSI-related symptoms might have been more likely to participate in post-discharge assessments than those with superficial SSIs or without symptoms, potentially leading to selection bias. Lastly, the reliance on clinical documentation to identify SSIs may have introduced information bias, as inconsistencies or omissions in records may have affected the accuracy of the data collected.

## 4. Methods

### 4.1. Study Design, Area, and Period

A longitudinal study was conducted between 1 June and 30 September 2022. The study focused on patients who had undergone surgery at JMC and were subsequently admitted to various wards, including orthopedics, general surgery, pediatric surgery, oral and maxillofacial surgery, maternity, and gynecology. JMC is a tertiary hospital which serves as a referral hospital to a total population of 20 million in southwest Ethiopia. Based on data from previous years, the hospital performs 5000 to 10,000 surgeries annually, including both elective and emergency procedures [18].

### 4.2. Study Population and Clinical Data Collection

A total of 1205 patients who had undergone various surgical procedures during the study period were recruited and included in the study consecutively. These participants were admitted to the specified wards at JMC and were then closely monitored daily throughout their hospital stay. Sociodemographic information was collected using structured questionnaires and clinical data was collected with direct observation or follow-up using standardized checklists adapted from WHO guidelines (Supplementary Table S1). Information about any medications administered to the patients after their surgical procedures, including the duration of administration, was extracted directly from the patients’ medication charts. Post-discharge, trained nurses contacted the study participants by telephone on days 7, 14, and 28 to conduct oral assessments for any signs of SSI. If an infection was suspected, patients were advised to visit the hospital for confirmation and to receive the necessary healthcare services.

Surgical site infection was defined as the occurrence of any superficial infection after surgery in the part of the body where the surgery took place, in the tissues under the skin (subcutaneous tissues) involved in the procedure during hospital stay or within 30 days post-discharge based on the WHO guideline [37]. The primary outcome of interest was the time to development of SSI during the participants’ hospital stay or within the 28-day follow-up period after discharge. The time to SSI was defined as the interval between the date of surgery and the date of SSI diagnosis, irrespective of whether the infection occurred during hospitalization or post-discharge. The duration of hospital stay was calculated as the total number of days the patient remained hospitalized from admission until discharge.

### 4.3. Specimen Collection and Bacterial Identification

Pus, abscess, or a pair of wound swab samples were collected aseptically from individuals with suspected clinical signs and symptoms of SSI. These samples were immediately transported to the microbiology laboratory using Amies transport media. Upon arrival, the samples were streaked onto 5% Colombia Sheep Blood and MacConkey Agars (Oxoid™, Basingstoke, Hampshire, UK) and then incubated aerobically at 35–37 °C for 24–48 h. If growth was observed, the isolates were subsequently identified using a battery of biochemical tests and then saved using storage media at JMC. Later, all the isolates were transported to Max von Pettenkofer Institute, Hospital Hygiene, and Medical Microbiology Laboratory in Munich, Germany, and re-identified with MALDI-TOF MS (Bruker, Ettlingen, Germany) technology.

### 4.4. Antimicrobial Susceptibility Testing

Antimicrobial susceptibility testing (AST) was conducted using the Kirby–Bauer disc diffusion technique for 25 antibiotics. To prepare the inoculum, a single colony was picked with a sterile cotton swab and suspended in sterile normal saline. The density of this suspension was then standardized to the 0.5 McFarland turbidity standard using DensiCHEK (Menarini Diagnostics, Florence, Italy). A sterile cotton swab was dipped into the standardized bacterial suspension, the excess fluid was removed by pressing the swab against the side of the tube, and it was then streaked on Muller Hinton Agar (Bio-Rad, Feldkirchen, Germany). Paper-impregnated antibiotic discs were then placed on the agar, and the plates were incubated at 35–37 °C for 16–18 h. The inhibition zone diameters around the antibiotic discs were read automatically using the ADAGIO™ system (Bio-Rad, Feldkirchen, Germany). The results were interpreted as susceptible, susceptible with increased exposure, or resistant according to the criteria set by the European Committee on Antimicrobial Susceptibility Testing (EUCAST, 2022) [38].

### 4.5. ESBL Screening

The phenotypic detection of ESBL production was performed for all Gram-negative isolates using a double disc synergy test (DDST). Ceftazidime (30 µg) and cefotaxime (30 µg) discs, along with an amoxicillin-clavulanic acid (10 µg) disc, were placed on Mueller–Hinton agar (Bio-Rad, Feldkirchen, Germany). The results were automatically interpreted by the ADAGIO system (Bio-Rad, Feldkirchen, Germany). Furthermore, the ESBL phenotypes were confirmed using Mast discs (Mast Diagnostica GmbH, Reinfeld, Germany) for all resistant isolates. The results were interpreted with the Mast discs combi D68C ESBL/AmpC calculator spreadsheet (Mast Diagnostica GmbH, Reinfeld, Germany) and reported as either negative or positive for ESBL and/or AmpC phenotypes.

### 4.6. Quality Assurance

To ensure sterility, 5% of each batch of the prepared media was incubated overnight at 35–37 °C aerobically, and any batches that showed evidence of bacterial growth were discarded. The quality of the antibiotic discs used for AST was also monitored weekly using the ATCC control strains including *P. aeruginosa* (ATCC-27853), *S. aureus* (ATCC-25923), and *E. coli* (ATCC-25922) (American Tissue Culture Collection, Manassas, VA, USA).

### 4.7. Data Processing and Analysis

Data was double-entered into Epi-Data version 4.6 and then exported to STATA version 17 for analysis. Descriptive statistics, including frequencies, proportions, medians and interquartile ranges, were calculated to present the demographic and clinical characteristics of the study participants and the length of hospital stay. Incidence of SSI was calculated per 1000 person-days and was reported at discharge, and days 7, 14, and 28 post-discharge with 95% Confidence Intervals (CIs). The Kaplan–Meier method was used to assess the median time to SSI. Cox-regression methods were used to assess differences associated with the surgical area, ward, and patient’s sex in developing the infection. Participants who were lost to follow-up for various reasons were censored in the analysis. The results of the Schoenfeld residual test indicate significant deviations from the proportional hazards assumption for the variable of hospital stay (*p* < 0.001). Consequently, the global test confirmed a significant violation of the proportional hazard assumption, leading us to use univariate and multivariate extended Cox-regression for time-varying covariates of hospital stay to identify risk factors associated with SSI. All relevant socio-demographic, clinical, physiological, and environmental variables were included in the multivariable extended Cox regression model to identify predictors of SSI. We then refined the final model by assessing changes in beta coefficients as variables were added or removed, comparing nested models based on the Akaike Information Criterion (AIC), and considering collinear relationships among included variables. The results were reported as crude hazard ratios (cHRs) from the univariate analysis and adjusted hazard ratios (aHRs) from the multivariate analysis, along with their corresponding 95% CIs. A *p*-value of less than 0.05 was considered statistically significant.

### 4.8. Ethical Consideration

Ethical clearance was obtained from the Institutional Review Board (IRB) of the Institute of Health, Jimma University (Reference number: IHRPGO/833). Written informed consent was obtained from each study participant. For child participants, assent was also sought in addition to parental/guardian consent. Confidentiality was ensured through anonymization of personal information. The culture and AST results were promptly communicated to the treating clinicians. Patients with culture-confirmed SSIs were then managed according to the hospital treatment protocol. Furthermore, patients who exhibited signs of SSIs during the post-discharge follow-up period based on the oral clinical assessments were advised to visit the medical center to receive appropriate healthcare.

## 5. Conclusions

This study highlights the substantial burden of SSIs, driven largely by Gram-negative bacteria in contrast to Western countries, with many strains exhibiting multidrug resistance. Key risk factors identified include older age, rural residence, male gender, emergency surgeries, certain anatomical sites, and hospital stay, which point to critical areas for targeted intervention. The findings stress the importance of robust infection prevention strategies, such as effective antimicrobial stewardship, environmental hygiene, and extended post-discharge surveillance. While the study provides valuable insights, its single-site focus and certain methodological limitations suggest that further research is needed to fully generalize the results.

## Figures and Tables

**Table 1:** Sociodemographic characteristics of study participants (n = 1205).

Variable		Frequency	Percent
**Sex**	Male	629	52.2
	Female	576	47.8
**Age**	<14	283	23.5
	15–24	220	18.3
	25–64	627	52.0
	>65	75	6.2
**Residence**	Urban	383	31.8
	Rural	822	68.2
**Wards**	Pediatric surgery	168	13.9
	OMFS	51	4.2
	General surgery	430	35.7
	Gynecology	136	11.3
	Orthopedics	270	22.4
	Maternity	150	12.4
**Area of surgery**	Head and neck	195	16.2
	Chest and back	59	4.9
	Upper limb	93	7.7
	Abdomen	615	51.0
	Perineum	65	5.4
	Lower limb	178	14.8
**Comorbid chronic disease**	Yes	37 *	3.1
	No	1168	96.9
**Indication for surgery**	Emergency	492	40.8
	Elective	713	59.2
**Mechanical bowl preparation**	Yes	326	27.1
	No	879	72.9
**Prophylaxis**	Yes	1187	98.5
	No	18	1.5
**Chlorhexidine cloth wash**	Yes	744	61.7
	No	461	38.3
**Skin preparation**	Yes	1200	99.6
	Unknown	5	0.4
**Chemical for skin**	Povidone Iodine	1188	99.0
	Absolute alcohol	10	0.8
	70% Alcohol	2	0.2

* Diabetes mellitus (n = 12), HIV (n = 5), Steroid drug users (n = 1), and Hypertension (n = 9), OMFS—Oral and Maxillofacial surgery, Prophylaxis antibiotics: ceftriaxone, ceftazidime, cefalexin, ampicillin, and metronidazole.

**Table 2:** Incidence of surgical site infections at various wards of JMC (n = 1205).

Variables		Incidence/1000 Person-Days					Time in Person-Days	Overall Incidence/1000 Person-Days [95% CI]
		At Discharge	Post-Discharge					
			Day 7	Day 14	Day 28	Total		
	Pediatrics (n = 168)	19.6	16.6	1.2	0	6.0	3304	13.6 [10.2, 18.3]
	OMFS (n = 51)	1.8	4.8	0	0	1.3	1298	1.5 [0.4, 6.2]
	Surgery (n = 430)	16.9	5.4	0.7	0.3	2.1	13,633	7.2 [5.9, 8.8]
**Wards**	Gyn & Obs (n = 136)	8.0	2.6	0	0	0.7	4691	3.2 [1.9, 5.3]
	Orthopedics (n = 270)	41.4	5.0	5.7	0	4.3	6743	20.8 [17.6, 24.5]
	Maternity (n = 150)	0	11.4	0.6	0	3.4	4197	2.9 [1.6, 5.1]
	Upper/lower limbs (n = 271)	0.7	1.9	0.3	0	0.6	9124	0.6 [0.3–1.5]
**Surgical**	Head/neck (n = 195)	38.8	6.2	3.2	0.3	3.6	4865	16.9 [13.6–20.9]
**area**	Abdomen (n = 615)	25.4	10.3	1.3	0.2	3.7	16,398	11.8 [10.3–13.6]
	Perineum/chest/back (n = 124)	17.3	5.2	2.7	0	2.8	3427	8.8 [6.1–12.5]
**Sex**	Female (n = 576)	12.0	5.9	1.3	0.2	2.4	17,684	5.4 [4.4–6.6]
	Male (n = 629)	27.5	8.0	1.6	0.1	3.1	16,159	13.4 [11.8–15.3]
**Total (n = 1205)**		**20.6**	**6.9**	**1.5**	**0.1**	**2.7**	**33,866**	**9.2 [8.3–10.3]**

**Key**: OMFS: oral and maxillofacial surgery; Gyn & Obs: gynecology and obstetrics.

**Table 3:** Univariate and multivariate extended Cox-regression analysis for risk factors associated with development of SSI up to 28 days post-hospital discharge at JMC (n = 1205).

Variables		SSI		cHR (95% CI)	*p*-Value	aHR (95% CI)	*p*-Value
		Yes	No				
**Age category**	≤14	81	202	1		1	
**(years)**	15–25	72	220	0.8 [0.57–1.07]	0.128	1.5 [1.00–2.33]	0.050 *
	26–50	112	356	0.7 [0.53–0.94]	0.018	1.8 [1.21–2.71]	0.004 *
	51–65	32	88	0.8 [0.52–1.17]	0.231	1.5 [0.89–2.41]	0.132
	>65	15	27	1.1 [0.63–1.89]	0.762	2.3 [1.21–4.24]	0.010 *
**Sex**	Female	95	481	1		1	
	Male	217	412	2.4 [1.90–3.09]	<0.001	1.7 [1.25–2.18]	<0.001 *
**Residence**	Urban	58	325	1		1	
	Rural	254	568	2.0 [1.53–2.71]	<0.001	1.4 [1.00–1.89]	0.049 *
	Maternity	12	138	1		1	
**Ward**	OMFS	2	49	0.6 [0.12–2.46]	0.435	1.9 [0.36–9.92]	0.451
	General surgery	98	332	2.6 [1.43–4.76]	0.002	7.3 [1.82–29.05]	0.005 *
	Pediatric surgery	45	123	4.2 [2.19–7.90]	<0.001	2.1 [0.72–6.27]	0.170
	Gynecology	15	121	1.2 [0.55–2.53]	0.673	1.3 [0.57–2.97]	0.540
	Orthopedics	140	130	7.4 [4.10–13.36]	<0.001	5.9 [2.35–14.55]	<0.001 *
**Area of surgery**	Upper and lower limb	6	265	1		1	
	Head and neck	82	113	23.8 [10.39–54.63]	<0.001	23.4 [10.10–54.34]	<0.001 *
	Abdomen	194	421	16.8 [7.47–37.99]	<0.001	16.2 [7.10–37.08]	<0.001 *
	Perineum, Chest, or Back	30	94	12.4 [5.15–29.77]	<0.001	11.0 [4.52–26.79]	<0.001 *
**Indication of**	Elective	205	508	1		1	
**surgery**	Emergency	107	385	0.8 [0.64–1.02]	0.072	1.9 [1.43–2.71]	<0.001 *
**Chlorhexidine cloth**	Yes	136	608	1		1	
**wash**	No	176	285	2.3 [2.11–3.30]	<0.001	1.5 [0.85–2.71]	0.154
**Pre-operative**	Yes	216	550	1		1	
**prophylaxis**	No	96	343	1.5 [1.19–1.92]	0.001	2.8 [0.79–9.80]	0.108
**Number of staff members involved in the surgery**		-		1.5 [1.11–2.11]	0.009	2.2 [1.29–3.65]	0.003 *
**Hospital stays as time-varying covariate (tvc)**		-		1.01 [1.00–1.02]	0.004	1.01 [1.00–1.02]	<0.005 *

**Key**: * *p*-value < 0.05.

**Table 4:** Antibiotic resistance patterns including intrinsic resistances among Gram-negative bacterial pathogens obtained from patients who developed SSI at Jimma Medical Center, Ethiopia.

Antibiotics	*E. coli* n (%)	*Acinetobacter* spp. n (%)	*Klebsiella* spp. n (%)	*Pseudomonas* spp. n (%)	*Proteus* spp. n (%)	*Enterobacter* spp. n (%)	Other GNB n (%)	Total n (%)
**AMP**	50 (89.3)	51 (100)	37 (100)	33 (100)	19 (79.2)	16 (100)	15 (93.8)	221 (94.8)
**PIP**	46 (82.1)	IE	37 (100)	33 (100)	17 (70.8)	16 (100)	12 (75)	161 (88.5)
**AMC**	44 (78.6)	51 (100)	33 (89.2)	33 (100)	16 (66.7)	16 (100)	11 (68.8)	204 (87.6)
**TZP**	37 (66.1)	IE	28 (75.7)	33 (100)	12 (50)	9 (56.3)	5 (31.3)	124 (68.1)
**CXM**	49 (87.5)	51 (100)	36 (97.3)	33 (100)	24 (100)	-	16 (100)	209 (96.3)
**CTX**	43 (76.8)	51 (100)	32 (86.5)	33 (100)	17 (70.8)	16 (100)	10 (62.5)	202 (86.7)
**CAZ**	39 (69.6)	-	30 (81.1)	24 (72.7)	8 (33.3)	15 (93.8)	9 (56.3)	125 (68.7)
**FEP**	41 (73.2)	-	31 (83.8)	33 (100)	16 (66.7)	14 (87.5)	7 (43.8)	142 (78)
**FOX**	24 (42.9)	-	7 (18.9)	-	1 (4.2)	16 (100)	10 (62.5)	58 (34.9)
**MEM**	5 (8.9)	26 (51)	7 (18.9)	9 (27.3)	0	2 (12.5)	2 (12.5)	51 (21.9)
**GM**	26 (46.4)	42 (82.4)	22 (75.7)	IE	17 (70.8)	11 (68.8)	5 (31.3)	129 (64.5)
**TM**	29 (51.8)	44 (86.3)	30 (81.1)	15 (45.5)	17 (70.8)	11 (68.8)	7 (43.8)	153 (65.7)
**AN**	7 (12.5)	11 (21.6)	6 (16.2)	3 (9.1)	4 (16.7)	0	4 (25)	35 (15)
**MXF**	49 (87.5)	-	29 (78.4)	-	16 (66.7)	11 (68.8)	7 (43.8)	103 (69.1)
**CIP**	44 (78.6)	35 (68.6)	29 (78.4)	24 (72.7)	12 (50)	8 (50)	8 (50)	160 (68.7)
**SXT**	42 (75)	32 (62.7)	30 (81.1)	-	17 (70.8)	13 (81.3)	7 (43.8)	141 (70.5)
**ESBL**	40 (71.4)	27 (52.9)	30 (81.1)	4 (12.1)	14 (58.3)	13 (81.3	7 (43.8)	135 (67.5)
**MDR**	44 (78.6)	44 (86.3)	31 (83.8)	28 (84.8)	15 (62.5)	13 (81.3	8 (50)	183 (78.5)

**Key**: AMP, ampicillin; PIP, piperacillin; AMC, amoxicillin + clavulanic acid; TZP, piperacillin + tazobactam; CXM, cefuroxime; CTX, cefotaxime; CAZ, ceftazidime; FEP, cefepime; FOX, cefoxitin; MEM, meropenem; GM, gentamicin; TM, tobramycin; AN, amikacin; MXF, moxifloxacin; CIP, ciprofloxacin; SXT, sulfamethoxazole + trimethoprim; **IE**: insufficient evidence; and “**-**” No breakpoints. **MDR**: resistant to at least one agent in three or more classes of antimicrobial agents.

## Data Availability

Access to the data will be granted in accordance with the policies of Jimma University, Health Institute and applicable ethical guidelines. Specific datasets may include clinical data, etiologies, and antimicrobial resistance patterns. Any additional conditions for accessing the data will be outlined in response to requests.

## Supplementary Materials

The following supporting information can be downloaded at: https://www.mdpi.com/article/10.3390/antibiotics15020201/s1, Table S1: Standardized checklists used to assess the development of SSIs during follow-up; Figure S1: Median hospital stays of study participants at JMC. Key: OMFS—Oral and Maxillofacial surgery; Gyn/Obs—Gynecology/Obstetrics.
