# Supplementary material for: Epidemiology of Surgical Site Infections: Incidence and Risk Factors at Jimma University Specialized and Comprehensive Hospital, Ethiopia
Source: Antibiotics (Basel). 2026 Feb 12;15(2):201. doi: 10.3390/antibiotics15020201 (PMC12937284; doi:10.3390/antibiotics15020201)
Supplement: Supplementary file 1 [file antibiotics-15-00201-s001.zip › antibiotics-4085963-supplementary.pdf]

Supplementary files

Supplementary Table S1: Standardized checklists used to assess the development of SSIs during follow-up.

Sr. No	Signs and symptoms of SSI	check all that apply
**1.**	Oozing or abscess drainage or visible soft tissue	
**2.**	Pain or tenderness at the surgical site	
**3.**	Hypothermia	
**4.**	Swelling or inflammation	
**5.**	Apnoea	
**6.**	Erythema or redness	
**7.**	Bradycardia	
**8.**	Lethargy	
**9.**	Fever (>38°C)	
**10.**	Cough	
**11.**	Nausea	
**12.**	Vomiting	
**13.**	Abscess	
**14.**	Dysuria	
**15.**	Incision deliberately opened/drained	
**16.**	Wound spontaneously dehisces	
**17.**	Surgeon's/ physician's diagnosis	

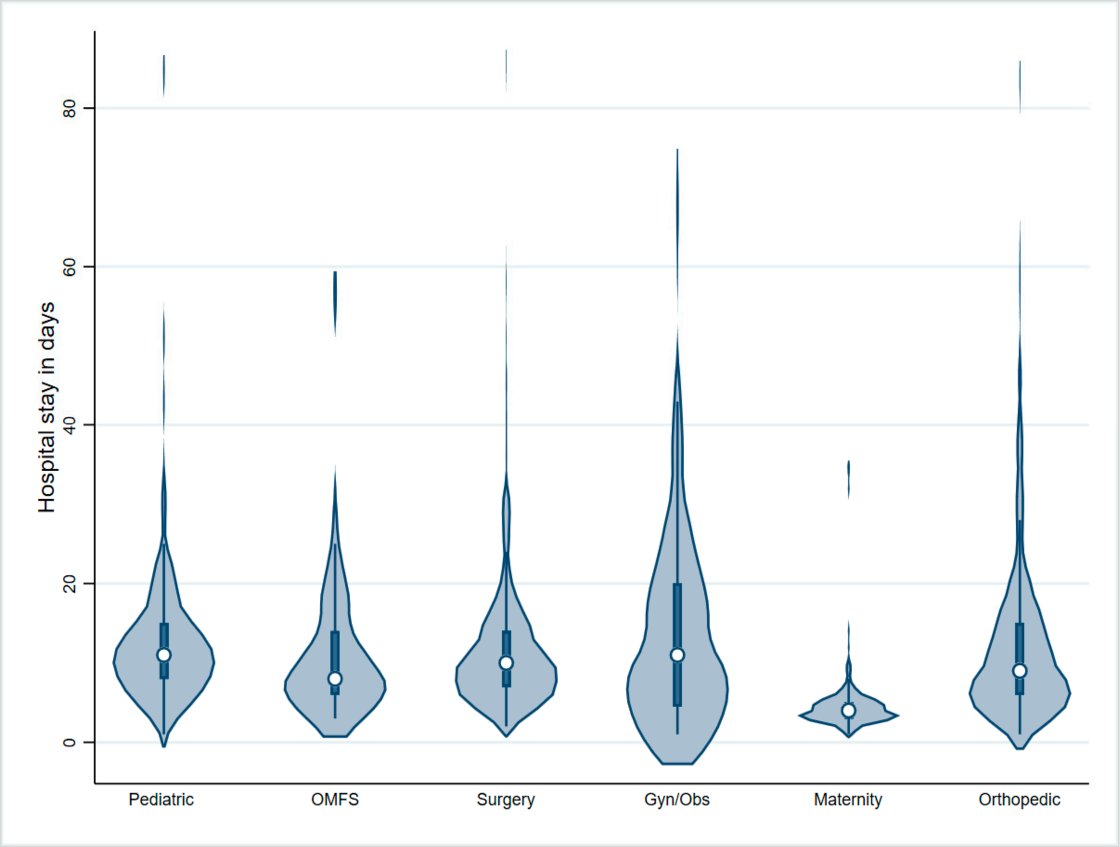

Supplementary Figure S1: Median hospital stays of study participants at JMC. **Key**: OMFS – Oral and Maxillofacial surgery; Gyn/Obs – Gynecology/Obstetrics
